# Procedural sedation by advanced practice providers in the emergency medical service in the Netherlands: a retrospective study

**DOI:** 10.1186/s13049-024-01207-z

**Published:** 2024-05-01

**Authors:** Risco van Vliet, Lennert Breedveld, Annemieke A.J. Heutinck, Bram H.A. Ockeloen, Arnoud W.J. van ’ Hof, Xavier R.J. Moors

**Affiliations:** 1 Regional Emergency Medical Services, RAV Brabant Midden-West-Noord, Gruttostraat 14, 5212VM, s-Hertogenbosch, the Netherlands; 2 Department of Cardiology, Maastricht University Medical Center, Maastricht, the Netherlands; 3 Department of Anesthesiology, Erasmus University Medical Center—Sophia Children’s Hospital, Rotterdam, the Netherlands; 4 Department of Cardiology, Zuyderland Medical Centre, Heerlen, the Netherlands; 5 Cardiovascular Research Instititute Maastricht (CARIM), Maastricht, the Netherlands

**Keywords:** Procedural sedation and analgesia, Ambulance, Emergency medical service, Advanced practice providers

## Abstract

**Background:**

Procedural sedation and analgesia (PSA) is a technique of administering sedatives to induce a state that allows the patient to tolerate painful procedures while maintaining cardiorespiratory function, a condition that is frequently desired prehospital. Non-physician prehospital clinicians often have a limited scope of practice when it comes to providing analgesia and sedation; sometimes resulting in a crew request for back-up from physician-staffed prehospital services.“. This is also the case if sedation is desirable. Advanced practice providers (APPs), who are legally authorized and trained to carry out this procedure, may be a solution when the physician-staffed service is not available or will not be available in time.

**Methods:**

The aim of this study is to gain insight in the circumstances in which an APP, working at the Dutch ambulance service “RAV Brabant MWN” from January 2019 to December 2022, uses propofol for PSA or to provide sedation. With this a retrospective observational document study we describe the characteristics of patients and ambulance runs and evaluates the interventions in terms of safety.

**Results:**

During the study period, the APPs administered propofol 157 times for 135 PSA and in 22 cases for providing sedation. The most common indication was musculoskeletal trauma such as fracture care or the reduction of joint dislocation. In 91% of the situations where propofol was used, the predetermined goal e.g. alignment of fractured extremity, repositioning of luxated joint or providing sedation the goal was achieved. There were 12 cases in which one or more adverse events were documented and all were successfully resolved by the APP. There were no cases of laryngospam, airway obstruction, nor anaphylaxis. None of the adverse events led to unexpected hospitalization or death.

**Conclusion:**

During the study period, the APPs performed 135 PSAs and provided 22 sedations. The success rate of predetermined goals was higher than that stated in the literature. Although there were a number of side effects, their incidences were lower than those reported in the literature, and these were resolved by the APP during the episode of care. Applying a PSA by an APP at the EMS “RAV Brabant MWN” appears to be safe with a high success rate.

**Supplementary Information:**

The online version contains supplementary material available at 10.1186/s13049-024-01207-z.

## Background

Procedural sedation and analgesia (PSA) is a technique for administering sedatives or dissociative agents with analgesics to induce a state that allows the patient to tolerate painful procedures while maintaining cardiorespiratory function [1]. PSA is performed for a variety of indications, such as alignment of fractured extremity or painful transfer, and is carefully conducted according to guidelines, Which are described for a clinical setting. As the administration of sedatives and opioids may result in cardiovascular collapse, respiratory depression, laryngospasm, or aspiration, careful hemodynamic monitoring with electrocardiography, heart rate, oxygen saturation and noninvasive blood pressure measurements is the standard of care [2, 3]. These guidelines are designed to provide frameworks for a PSA that improve patient safety. However, this puts a restriction on performing a PSA only in the clinic. To extend it beyond the operating room, these guidelines have been further developed in the Netherlands [1]. Although these guidelines relate primarily to procedures and operations that take place within a hospital, the recommendations are also applicable to PSAs outside hospitals, for example in dentistry and independent treatment centers. This development of the guidelines offers an opportunity for the use of PSA, by non-physicians in the acute prehospital setting. Our review of the medical literature revealed limited evidence on this topic. Attempts to introduce these skills to the general paramedic prehospital care provider were made in San Diego, but showed unfavorable results for induction of anesthesia. However, training a small group of prehospital caregivers has been shown to improve outcomes for trauma patients [4]. Within the Dutch setting, this topic has never been studied and the outcomes are invisible and unclear. Could this limited evidence also be visible in the Dutch acute setting?.

Regular ambulances in the Netherlands are staffed with a driver and an ambulance nurse. A registered nurse can qualify as an ambulance nurse after completing a specific national training course. Dutch ambulance care professionals have functional autonomy within the framework of the national emergency medical service (EMS) standard. This standard includes 113 flowcharts with decision-making strategies for the diagnosis and treatment of symptoms of 15 diagnosis groups (e.g., airway, cardiology, internal medicine and trauma care) [5]. The flowchart for analgesia allows the ambulance nurse to use paracetamol, fentanyl, and ketamine. The authorized dosage has an analgesic but not a sedative effect. Sometimes the nature of a condition or the status of a patient requires sedation or anesthetics; however, The existing flowchart does not allow the addition of sedation and is sometimes inadequate.

RAV Brabant MWN (regional ambulance service for Brabant Mid-West-North), an EMS located in the south of the Netherlands, introduced advanced practice providers (APPs) in January 2019 [6]. APPs are qualified nurse practitioners or physician assistants working for the Dutch EMS RAV Brabant MWN. They are either postgraduate Master of Science-trained nurse practitioners or postgraduate Master of Science-trained physician assistants (Netherlands/European Qualifications Framework, Level 7). Before working for the EMS, they were educated as registered nurses (Netherlands/European Qualifications Framework, Level 4 or 5), with supplementary training as intensive care nurses, emergency nurses, or anesthesia nurses. They attend an annual training program and have a legal obligation to obtain a certain number of training points. PSA training consists of a theoretical part and a practical part, both of which end with an examination. Current guidelines [1, 13, 14], both national and international, are used for this training. The APP works independently, but when performing PSAs, they are always supported by a full ambulance team trained in advanced life support. In addition, there is always an opportunity for telephone supervision by one of the medical managers. A medical manager is an emergency physician or anesthesiologist who is responsible for the medical implementation of this EMS agency. For the first five individual PSAs done by an APP, the plan and goal are discussed in advance with the medical manager. Annually, all PSAs are evaluated by an anesthesiologist, and APPs undergo retraining. Patients who are ASA 3 or 4 require consultation with the on duty medical manager. If quality and safety requirements are met, PSA may be performed by APP.

An APP has the authority to use other guidelines in addition to the national EMS standard. Following changes to Dutch healthcare legislation in 2018, nurse practitioners and physician assistants in this EMS are independent prescribers and they may for instance perform cardioversion and certain simple surgical procedures including thoracostomy on their own previously, reserved for doctors, dentists, and midwives, including PSA [6, 7]. This change allowed for new treatment options not previously allowed by the national protocol. Before the initiation of the APP service, the ambulance crew had to request back-up from a physicain-staffed serviceA (i.e. HEMS) if they felt that a patient may benefit from a PSA. The APP can be a solution if the helicopter EMS is unavailable or does not arrive in time. An additional benefit of APP PSA is that it has introduced propofol which allows the opportunity to use this agent for sedation. The need for sedation occurs for such things as intractable seizures, agitation after return of circulation in cardiac arrest and for behavioral emergencies with severe agitation and delirium. Normal EMS standards advise Midazolam having the disadvantages difficultly controlling dosage and the longer duration of action when this is not desirable. The authors found limited evidence, that PSA is performed in ambulance care by non-physician EMS professionals, or was there no description of which professional had performed the PSA [4, 7]. The purpose of this study is to describe the use and safety profile of PSA by Dutch APPs.

## Methods

### Aim

The aim of this study is to [1] describe the characteristics of patients and ambulance runs in situations where an APP uses propofol for a PSA or to provide sedation and [2] evaluate these interventions in terms of safety.

To achieve this aim, the following variables and outcomes will be collected. see Table 1.

**Table 1:** Variables and the associated outcomes

Variables	outcomes	
Characteristics of the patients and ambulance runs	demographic data	Age Sex Body mass index American Society of Anesthesiologists [ASA] score
	process data	Time Dispatch urgencies Routes
	medical data	Initial dispatch chief complain On-scene diagnosis Medication used
Safety	number of adverse events	Airway obstruction Apnea (> 20 s) Hypoxia (oxygen saturation < 90% for > 60 s) Hypotension (systolic blood pressure < 90 mmHg) Bradycardia (< 50/min) Tachycardia (> 100/min) Agitation Aspiration Hospital admission due to PSA

To classify the adverse event, associated interventions and or the outcome of the adverse event, the World SIVA adverse sedation reporting tool was applied retrospectively [8, 9]. See Table 2.

**Table 2:** SIVA variables and outcome

Minimal risk descriptors	Minor risk descriptors	Sentinel risk descriptors	
Vomiting Subclinical respiratory depression Muscle rigidity, myoclonus Hypersalivation Paradoxical response Recovery agitation Prolonged recovery	Oxygen desaturation (75–90%) for < 60 s Apnea, not prolonged Airway obstruction Failed sedation Allergic reaction without anaphylaxis Bradycardia Tachycardia Hypotension Hypertension Seizure	Oxygen desaturation, severe (< 75% at any time) or prolonged (< 90% for > 60 s) Apnea, prolonged (> 60 s) Cardiovascular collapse/ shock Cardiac arrest / Absent pulse	
the INTERVENTIONS performed to treat the adverse events(s).			
**Minimal risk**	**Minor risk**	**Moderate risk**	**Sentinal intervention**
No intervention performed Administration of: Additional sedative(s) Antiemetic Antihistamine	Airway repositioning Tactile stimulation or the administration of: Supplemental oxygen, new or increased Antisialogogue	Bag valve mask assisted ventilation Laryngeal mask airway Oral/nasal airway CPAP or the administration of: Reversal agents Rapid i.v. fluids Anticonvulsant i.v.	Chest compressions Tracheal intubation or the administration of: Neuromuscular block Pressor / epinephrine Atropine to threat bradycardia
**Outcome of the adverse event**			
**Minimal risk outcome**	**Moderate risk outcome**	**Sentinel outcome**	
No adverse outcome	Unplanned hospitalization or escalation or care	Death Permanent neurological deficit Pulmonary aspiration syndrome	

If there are any adverse event outcome is checked in the Sentinel columns above, then this is a Sentinel adverse event. The same is used for the Moderate risk adverse event and Minimal risk adverse event. Last described is whether the predetermined goal was achieved.

### Design

This is a retrospective observational study of all patients who received propofol administered by an APP working at the Dutch EMS RAV Brabant MWN from January 2019 to December 2022.

### Setting

The APPs works for an EMS organization in the southern part of the Netherlands. This region has a population of approximately 1.8 million people. The EMS covers three main urban areas: Tilburg, Breda, and ‘s-Hertogenbosch. The region, RAV Brabant MWN, has 86 ambulances and 25 ambulance stations. In 2019, there were 139,271 ambulance runs in the region [10].

At the start of this study eight APPs were only stationed in Tilburg and were on call between 10 AM and 6 PM. After one year, the shifts were expanded to day and evening shifts (beginning at 7 AM and 10 PM). In the third year, coverage was expanded to the ‘s-Hertogenbosch and Breda regions, resulting in five different shifts. The 15 APPs alternately divide into three day shifts and 2 evening shifts per day, in which an APP is available in different three regions [11]. 

### Procedural sedation and analgesia

The definition of PSA used in this EMS is largely consistent with what has been described in the literature, namely; a technique for administering sedative or dissociative agents with analgesics to induce a state that enables the patient to tolerate painful procedures while preserving cardiorespiratory function. In addition, propofol can be used when sedation is needed to treat convulsions, post ROSC in OHCA and severe agitation with delirium or similar when only agitation needs to be controlled. The intervention was performed according to the latest guidelines [1]. A preprocedural screening was performed using a standardized PSA registration form. Information recorded included ambulance run number, informed consent, height and weight, ASA classification, medical history, allergies, expected airway difficulties, and fasting state. Vital signs were measured at regular intervals, during and after the procedure, until the patient was fully awake. The vital signs included blood pressure, heart rate, pulse oximetry and end-tidal CO_2_. When esketamine was used, the dissociative state was registered (*yes*/*no*). Airway rescue equipment was available within the hand’s reach, and full resuscitation equipment was readily available. The patients received a sedative or analgesic depending on the situation. Drugs that can be used are single doses or combinations of propofol, esketamine, fentanyl, and midazolam. After propofol was administered for a PSA or to provide sedation, the APP registered if the procedure for which the PSA was needed was successful completed by describing if the predetermined goals were met. All adverse event occurred and all appropriate intervention were described.

### Data

From January 2019 to December 2022, data were collected from all ambulance runs where an APP used PSA or provide sedation. The data were collected from three sources: [1] emergency medical dispatch center database [2], regular ambulance run sheets, and [3] a database for the PSA. Each ambulance run was stored in an EMS database and has a unique identification number, which can be used to connect the three data sources at the patient level and guarantees anonymity.

### Data analysis

The data were analyzed with SPSS version 25.0 (or higher if available). No power calculation was made. The data was entered into the database by one researcher, which was checked by a second researcher, both are postgraduate Master of Science-trained nurse practitioners (RvV, LB). If discrepancies were found, the data were adjusted in mutual discussion and with checking of the source documents. The analysis was also calculated individually by two researchers and then merged and described (RvV, BO).

## Results

### Demographic

During the inclusion period, the APP administered 157 times Propofol. This was in 135 for PSA and in 22 cases for providing sedation. Table 3 shows how the numbers are distributed by intervention per year.

**Table 3:** Distribution per intervention per year

Year	PSA n	Sedation n	%
2019	30	1	19.7
2020	13	2	9.6
2021	27	7	21.7
2022	65	12	49.0
Total	135	22	100

The characteristics of the patients and ambulance runs where where an APP uses propofol for a PSA or provide sedation was applied are shown in Table 4. Propofol was used more often for women than for men (56.1% versus 43.9%). The mean age was 62.3 years and the median was 71, with 69.5% of the patients over 50 years of age and 5.8% under 18, which is considered nonadult in the Netherlands. Out of the 157 patients, 57.3% had an ASA score of 1, and 10.8% scored higher than ASA 2. Moreover, 84.1% had a healthy body mass index, and 10.2% were Overweight.

**Table 4:** Characteristics of the patients and ambulance runs

Sex	n	%
Male	69	43.9
Female	88	56.1
Total	157	
Age group (years)	*n*	%
0–10	2	1.35
11–17	7	4.5
18–50	33	21.0
51–75	56	35.7
> 75	53	33.8
Missing data	6	3.8
Age	Years	
Mean	62.3	
Median	71	
Range	95	
ASA class	*n*	%
ASA 1	90	57.3
ASA 2	50	31.8
ASA 3	13	8.3
ASA 4	4	2.5
Body mass index	*n*	%
Underweight	6	3.8
Healthy weight	132	85.7
Overweight	16	10.4
Missing data	3	1.9
Total	157	100

### Process data

Eight (2.5%) patients were not transported to the hospital after administration of Propofol. In 125 (79.6%) of the cases, the APP transferred the care to the ambulance crew. In the other cases (*n* = 28), the APP stayed in the ambulance to continue the patient’s care. Table 5 shows that 75.2% of the consultations in which Propofol was used was applied after a citizen alert via national emergency number (112), 17.2% were given a PSA or provided sedation at the request of an ambulance team, and 29.3% had the highest ambulance run urgency (A1: blue lights and siren).

**Table 5:** Process data

	n	%
Treated by an APP and transferred to ambulance crew	125	79.6
Medical assistant	28	17.8
Treatment at the scene	4	2.5
Means of requesting help	*n*	%
National emergency number	118	75.2
General practitioner	9	5.7
Ambulance	27	17.2
Police	1	0.6
Missing data	2	1.2
Dispatch urgency		
A1: a life-threatening situation. The ambulance uses sirens and flashing lights and must arrive at the scene within 15 min of being dispatched.	46	29.3
A2: a non-life-threatening situation but nonetheless urgent. The ambulance must arrive at the scene within 30 min.	106	67.5
B: plannable care. The ambulance is dispatched without a time limit.	5	3.2

APP; Advanced practice providers

### Medical data

Table 6 shows the initial dispatch chief complaint and on-scene diagnoses of patients to whom PSA or sedation was applied and the treatment given. The top three initial dispatch chief complaints were fall/fall from height (*n* = 63, 40.1%), traumatic injury (*n* = 50, 31.8%), and traffic accidents/transport accidents (*n* = 16, 10.2%). Most patients had an on-scene diagnosis related to traumatology or a surgery condition (*n* = 120, 76.4%). Table 7 summarizes which medication was used.

**Table 6:** Medical data

Initial dispatch chief complaint	n	%
Fall	63	40.1
Traumatic injury	50	31.8
Traffic accident	16	10.2
Cardiac arrest	7	4.5
Sick call or undefined complaint	6	3.8
Chest pain	2	1.3
Seizure	2	1.3
Heart problem	2	1.3
Overdose	2	1.3
Fainting	2	1.3
Unknown problem	2	1.3
Abdominal pain	1	0.6
Choking	1	0.6
Industrial accident	1	0.6
On-scene diagnosis	*n*	%
Fracture of lower extremity	50	31.8
Luxation of hip	37	23.6
Fracture of hip	17	10.8
Fracture of upper extremity	16	10.2
Sedation after OHCA	10	6.4
Luxation of shoulder	9	5.7
Luxation of ankle	6	3.8
Neurological problem	5	3.2
Treatment of insufficient heart rhythm	3	1.9
Convulsions	2	1.3
Severe agitation with delirium or similar	2	1.3
Treatment under procedural sedation and analgesia	*n*	%
Alignment of fractured extremity	83	52.9
Reposition of dislocated joint	52	33.1
Sedation after OHCA	10	6.4
Sedation	9	5.7
Cardioversion	3	1.9

OHCA; Out of hospital cardic arrest

**Table 7:** Medication given

	n	%
Propofol	157	100
Combination		
Propofol with fentanyl	82	52.2
Propofol with ketamine	90	57.3
Propofol with midazolam	5	3.1
Propofol with fentanyl and ketamine	37	23.5

### Safety

Of the 157 cases in which a PSA was administered or sedation was provided, telephone contact was made with a physician for supervision and to discuss the reason, purpose and method in 68 (43.3%) cases. In 12 cases (7.6%), one or more adverse events occurred during or shortly after administrating Propofol. See Table 8.

**Table 8:** Adverse events per individual cases

Adverse event individual cases	Apnea > 30 s	Oxygen saturation < 90% and > 60 s	Vomiting	Bradycardia (< 50/min)	Tachycardia (> 100/min)	Aspiration
1	1	1		1		
1	1					
1	1					
1	1					
1	1					
1	1					
1		1				
1		1				
1			1			
1					1	
1					1	
1			1			1

To provide more clarity on the adverse events, the World SIVA adverse sedation event-reporting tool was used [12].

According to Table 9, there were two (1.2%) minimal risk and twelve (7.6%) minor risk adverse events. Most cases required minimal to no interventions: 149 (94.9%) minimal (no intervention, additional sedatives, antiemetics or antihistamines); five (3.1%) mild (airway repositioning, tactile stimulation, supplemental oxygen or antisialagogue); and three (1.9%) moderate (ventilation with ventilation balloons, laryngeal mask, oral/nasal airway procedure, continuous positive airway pressure, reversal agents, rapid intravenous fluids or intravenous anticonvulsant). All resulted in a minimal risk outcome.

When severity rating was applied, there were 147 (93.6%) minimal risk adverse events, seven (4.4%) minor risk events and three (1.9%) moderate risk events.

**Table 9:** World SIVA adverse sedation event reporting tool

Step 1: Was there one or more adverse events associated with this sedation encounter?							
*N* = 12							
Step 2: DESCRIBE the adverse events(s). Check all that apply.							
*Minimal risk descriptors* *n* = 2 Vomiting (*n* = 1aspirated)		*Minor risk descriptors* *n* = 3 Oxygen desaturation (75–90%) < 60 s (*n* = 1 apnea, not prolonged) *n* = 5 Apnea, not prolonged *n* = 2 Tachycardia *n* = 1 Bradycardia *n* = 1 Hypotension				*Sentinel risk descriptors*	
Step 3: INTERVENTIONS performed to treat the adverse events(s).							
*Minimal risk* *n* = 149 No intervention performed		*Minor risk* *n* = 5 Airway repositioning Bag valve mask assisted ventilation. Tactile stimulation or Supplemental oxygen, new or increased	*Moderate risk* *N* = 3 Bag valve mask assisted ventilation		*Sentinel intervention*		
Step 4: OUTCOME of the adverse events(s)							
*Minimal risk outcome* *n* = 157 No adverse outcome	*Moderate risk outcome*			*Sentinel outcome*			
Step 5: SEVERITY rating to the adverse event(s) associated with sedation.							
*Minimal risk adverse event.* *N* = 147		*Minor risk adverse event.* *N* = 7	*Moderate risk adverse event.* *N* = 3		*Sentinel adverse event.* *N* = 0		

Secondary safety outcomes were success rate of achieving the predetermined goals of the procedure (Table 10). The APPs reported in 135 patient records that the PSA achieved the predetermined goal in 122 (90.3%) cases and failed in 13 (8.2%) cases. The failures included four (2.5%) cases of alignment of a fractured extremity and nine (5.7%) of repositioning of luxated joints. In 22 cases, the goal was sedation, for example, after an OHCA or in preparation for cardioversion. The goal was achieved in all of these cases.

**Table 10:** Success rate of achieving the predetermined goals of the procedure

	Goal met, n (%)	Goal not met, n (%)
Alignment of fractured extremity	79 (50.3)	4 (2.5)
Repositioning of luxated joint	43 (27.3)	9 (5.7)
Sedation	9	0
Sedation after OHCA	10	0
Cardioversion	3	0
Total	144(91.7)	13 (8.2)

OHCA; Out of hospital cardic arrest

Four patients were not transported to a hospital after PSA. A customized plan was made on site that prioritized comfort and effective care. One situation involved an elderly patient in a nursing home. A customized plan was agreed upon between the APP, geriatrician, and family. In two cases, a hip luxation was set at home, and, in consultation with the orthopedist, an outpatient appointment was made for a later date. Another case involved a young patient with a habitual shoulder luxation who, after repositioning, was also seen by his orthopedist through an outpatient clinic. Although there have been relatively few cases of APPs applying PSAs with results in non-conveyance, this approach may be the start of a new safe applied care pathway that matches the patient’s needs and could have a positive effect on EMS or emergency department resources and patient safety [16].

In 68 cases, the medical director was contacted by telephone. The reason, purpose, and method of the PSA were discussed before the PSA was used. Most of them were cases with an ASA classification ≤ 2. Of the ASA-4 patients, there was no telephone contact in two of the four ASA-4 patients, however, one patient received sedation after OHCA, and the other had an on-site consultation with the helicopter EMS physician. Of the 13 ASA-3 patients, seven had a retrospective review in which an incorrect ASA classification was calculated. This was another indication of deficiencies in the patient data.

## Discussion

The data show that in 157 individual cases Propofol was used by the APP. 135 times for a PSA and 22 times to provide sedation. These cases were spread over four years, from 2019 to 2022. with the incidence increasing toward the later years. A possible explanation is that assigning appropriate ambulance runs to an APP has proven to be challenging for the dispatch operator [6]. Initially, it seems to have been almost a coincidence that an APP was assigned to a patient in need of a PSA; in subsequent years, however, with more awareness of the competencies involved, APPs were more often tasked or requested for medical assistance. The growth in 2022 can be explained by the expansion of the regions covered and the hours worked. The expectation is that the total number of ambulance runs can increase if there is proper assignment of APP’s from the dispatch center. As an example, in 2020 there were approximately 25,000 patients with traumatology as a working diagnosis, which was 17% of all ambulance deployments at this EMS agency [13]. In a large number of these cases, a PSA could be an advantage in managing acute pain during a painful procedure, which would have particular benefits for elderly patients [14].

Most PSAs (90%) were used for patients with extremity injuries. The most common injuries were fractures or luxation of joints. This is consistent with previous studies in which PSAs were used in support of limb realignment and splinting [7, 15]. However, we found a higher success rate than a previous study [7]. As described in the background, the EMS professional also has the option of using paracetamol, fentanyl or ketamine. The existing flow chart does not allow for effective management of some extremity fractures resulting in re-dosing which can be accompanied by side effects, e.g. nausea, drowsiness and hallucinations, anxiety, disorientation. And may contribute to failure to achieve the clinical goal. The proposed advantage of a PSA is that it has a predictable, potent and short-lasting effect, so the patient wakes up shortly after the procedure and with less adverse effects. This was supported by the results of our study.

One or more adverse events occurred in 12 (7.6%) of the 157 cases. This percentage is less than the percentage reported in the literature, which is usually between 8% and 11% [14, 16, 17] or even higher [18], with the most common occurrence being hypoxia. All occurrences could be resolved by the APP at the scene. The frequency of events requiring APP-performed PSA was relatively small. Therefore there were limited clinical opportunities for each APP to perform PSA which raises the issue of maintenance of clinical competency. To remain sufficiently competent the APP had a PSA training once a year and individual cases were discussed among the group once a year at another training day. The expectation is that as awareness increases at the dispatch center, the number of PSAs will increase. By conducting this study, the researchers realize that there are many differences EMS systems worldwide with many different levels of training and tasks performed. As a result, the importance of this study may be valued differently. In the Netherlands, this situation is still very unique and needs further exploration. Therefore, we think this study is of great importance and could serve as an inspiration for other EMS services to eventually offer good care to the critically ill prehospital patient.

### Study limitations

This retrospective study obtained most of its data written patient care reports and therefore relied heavily on the accuracy and thoroughness of the documentation. Consequently, there is a chance of over or under reporting. Not all patient data were complete or clear. The data from the dispatch database, the ambulance run sheet, and the additional PSA database sometimes lacked an identical identification number, this required manually checking all cases by patient name, date and time of care so that all individual databases could be properly linked. As a result, time was lost organizing these data. In some cases the data were incomplete and had to be retrieved retrospectively from the APP. This often involved the ambulance run number, time or dates. Again, this was time consuming but did not result in missing data. Despite the fact that filling in missing data afterwards could lead to reporting bias, the authors deliberately chose to do so. Because the number of cases was so low and could now be included in the calculation. An improvement in the data management system is recommended to overcome this obstacle [19] but has not yet been implemented.

The overall number of uses of propofol is small. This study has the potential for having missed seeing adverse events simply because of the limited sample size and their overall infrequency. Further research with a lager sample size could help buttress our findings.

Even though this EMS covers a large region, the APP only work in a small urban area, so the results may not be representative for the whole region. It would be desirable to expand the study to include non-urban areas to confirm the generalizability of the findings.

## Conclusion

This article provides the first description of administrating of Propofol by nonphysicians in a Dutch EMS agency. During the study period, the APPs administered propofol 157 times for 135 PSA and in 22 cases for providing sedation. The predetermined goals were to facilitate limb realignment, splinting or sedation. The success rate was higher than that stated in the literature. Although there were a number of side effects they were lower than those reported in the literature, and these were resolved by the APP during the eposide of care. Applying a PSA by an APP at the EMS “RAV Brabant MWN” appears to be safe with a high success rate.

### Electronic supplementary material

Below is the link to the electronic supplementary material.

Supplementary Material 1

## Data Availability

The datasets used and/or analyzed during the current study are available from the corresponding author on reasonable request.

## Abbreviations

APP: Advanced practice provider
ASA: American Society of Anesthesiologists
EMS: Emergency medical service
OHCA: Out-of-hospital cardiac arrest
PSA: Procedural sedation and analgesia
ROSC: Return of spontaneous circulation (after ROSC)
RAV: Brabant MWN Regional ambulance service for Brabant Mid-West-North
