# Supplementary material for: Procedural sedation by advanced practice providers in the emergency medical service in the Netherlands: a retrospective study
Source: Scand J Trauma Resusc Emerg Med. 2024 May 1;32:39. doi: 10.1186/s13049-024-01207-z (PMC11064379; doi:10.1186/s13049-024-01207-z)
Supplement: Supplementary file 1 — Supplementary Material 1 [file 13049_2024_1207_MOESM1_ESM.docx]

View Letter

Date:	27 Feb 2024

To:	"Risco van Vliet" r.vliet@ravbrabantmwn.nl

cc:	marius.rehn@norskluftambulanse.no

From:	"Scandinavian Journal of Trauma, Resuscitation and Emergency Medicine" kristoval.ferrer@springer.com

Subject:	Your submission to Scandinavian Journal of Trauma, Resuscitation and Emergency Medicine - STRE-D-23-00353R1

STRE-D-23-00353R1

Procedural sedation by advanced practice providers in the emergency medical service in the Netherlands: A retrospective study

Risco van Vliet; Lennert Breedveld; Annemiek Heutinck; Bram Ockeloen; Arnoud van 't hof; Xavier Moors

Scandinavian Journal of Trauma, Resuscitation and Emergency Medicine

Dear Mr Vliet,

Your revised manuscript 'Procedural sedation by advanced practice providers in the emergency medical service in the Netherlands: A retrospective study' (STRE-D-23-00353R1) has been assessed by our reviewers. It is still of interest,

but unfortunately we are unable to consider it for publication in its current form. The reviewers have raised a number of points which we believe would improve the manuscript and may allow a revised version to be published in Scandinavian Journal of Trauma, Resuscitation and Emergency Medicine.

Because the reviewers did not view your manuscript in the same way, I have also reviewed it myself.

Please see the attached pdf for my comments and suggestions.

Reviewer reports:

Reviewer #1: Thank you for the changes made and pursuing publication, and for letting me review the new manuscript. I note that you have made positive changes to the language used and the manuscript reads much better in your second submission.

My position on this article as a potentially important piece in the literature remains steadfast but my view after assessing then resubmission is that some further changes are required for it to publish. Please see below for feedback to consider. I hope you choose to continue developing this manuscript and chase publication with SJTREM.

dear reviewer #1

Thank you again for your thorough review and your encouragement to improve this manuscript. Below, point by point, is our response to your comments.

Reviewers comments:

*- Include the aim in the abstract*

the aim is now more clearly described in the abstract under method

*- In the abstract, the 'Conclusion' repeats the 'Results' ('Results reads OK, but conclusion could be more pertinent - how can this study impact practice?). Ensure abstract is representative of article.*

has been adapted and written down more directly

*- Lines 69 and 70: Unclear which settings the authors refer to where PSA is used/not used. It reads as if there is no literature on PSA about prehospital care. Which databases did you search? Is this limited to Dutch literature?* short explanation that it is mainly about the clinsic setting has now been added line 73

*See studies by Vopelius-Feldt et al (2014, doi: 10.1136/emermed-2013-202895) and Bredmose et al (2009, doi: 10.1136/emj.2007.052753) for examples of sedation used prehospital by others than physicians. For publication in an international journal, I would expect to see this mentioned and considered.* Thank you for this valuable addition. We have now incorporated this throughout the manuscript. The fact remains that there is very little literature available and the different stettings and qualifications are very different. This makes it difficult to draw a conclusion.

Linked with: last few sentences on page 8 (formatting makes line numbering ambiguous): There is a reference to literature stating PSA is performed globally but this is not cited. Indeed the line numbers have shifted due to the adjustments, your comment is now processed at line 135.

*- PSA still applied to seizures, agitation, and ROSC, which in the anglophone world are separate treatment pathways with separate expectations of monitoring and urgency. A PSA is seen as elective option when the expertise is available, whilst management of convulsions, severe agitations in a person posing threat to self and others, or post-ROSC are considered medical emergencies. This clouds the data and acknowledging this would be good practice.*

In our opinion, a PSA is the administration of combination of different drugs. A sedative and an analgesic. irrespective of whether this is done electively or acutely. Worldwide, there are many differences in EMS systems and the tasks they perform. Within the Netherlands, this performance by non-physicians is so unique that we still find it important to write this manuscript. Following your earlier comments, we have now described PSA and sedation more clearly in the purpose and situation in which each is applied. We believe that it is now clearer how and when we applied it. Despite the global differences, we have provided an initial insight into our Dutch situation. we hope this is clear to the reader.

*- Secondary outcome "level of success" - how is this defined or evaluated?* by describing if the predetermined goals were met. 189

*- Lines 146-148 "master of science-trained" could be exchanged e.g., "postgraduate qualification"* thank you for this addition, has now been updated at several points in the manuscript

- Line 169 describing esketamine dissociation scoring could be moved to "data" or at least placed after first describing the relevant sedation agents

Unfortunately, no scoring instrument is used in tracking the degree of disiosiation. For that also not included in this study. But a good observation that we will incorporate into our current reporting structure

*- Line 186 was data analysed across several platforms or by multiple people? It says SPSS 25 was used or higher if available. Clarification on who performed the analysis and their qualification to do so could be helpful* adjusted line 202 -204

*- Line 200, consider other terminology than 'obese'. I am aware this is widely used in continental Europe without negative connotation but this has garnered unfavourable associations in the Anglophone world*

Never realised that this term could be included offensively elsewhere. For that, modified the term in Overweight on line 118 and in the table

*- The reporting of the SIVA score still appears to be adapted to the authors' narrative of PSA as safe. Acknowledging that whilst SIVA outcomes never escalated beyond minimal, there were some sentinel events reported in the matrix clouded by the reporting.*

Applying the SIVA tool, we considered that this event belonged to the "Minor risk descriptors". Therefore, we also did not report it as sentinal.

*- Why do the authors think PSA was used more frequently in women than men? It is known that the burden of trauma is higher in men than women and the study has a high incidence of traumatic injuries, so what could have caused this demographic shift in your study? E.g., could this be cultural/impartial treatment, or was the severity of injury higher in men and so they required more invasive management beyond PSA (e.g., RSI or resuscitation). Worth discussing as this is an interesting result.*

It is indeed a special fact. However, because the numbers are low, we have not done anything with this result. We think this is more a coincidence than an explanation can be given. Only when the APP is fully integrated into the organization and the APP is present in most case studies can this fact be investigated. Thank you for noticing this fact.

General comments:

*- How was missing data managed?*

Dit werd beschreven in de verschillende tabellen. De invloed van deze missing data was erg laag en is niet meegenomen in de verschillende berekeningen.

*I appreciate your effort in contributing to prehospital research and your persistence in pursuing publication. The study is interesting but also holds a range of limitations due to its design. It is therefore important to ensure it is otherwise rigid in its reporting of context and limitation so as not to confuse the reader about its importance.*

Thank you for this comment, this is indeed true for that we added a comment to the discussion at lines 333 - 363

Reviewer #2: Thank you for allowing me to review the revised manuscript.

The authors have provided a thorough response to the raised comments and I found the manuscript much clearer. In my opinion, the revised manuscript is acceptable for publication.

Well done, I look forward to reading this article in SJTREM in the near future.
